# Prevalence and clinical relevance of corneal astigmatism in cataract surgery candidates: a southern Italian cohort study

**DOI:** 10.3389/fmed.2025.1696340

**Published:** 2026-01-21

**Authors:** Francesco D’Oria, Pasquale Puzo, Ali Nowrouzi, Francesco Boscia, Giovanni Alessio

**Affiliations:** 1 Section of Ophthalmology, Department of Translational Biomedicine and Neuroscience, University of Bari, Bari, Italy; 2 Department of Ophthalmology, Hospital Quirónsalud Marbella, Marbella, Spain

**Keywords:** axial length, cataract surgery, corneal astigmatism, ocular biometry, toric IOL

## Abstract

**Background:**

Corneal astigmatism is a major determinant of visual outcomes after cataract surgery, and its accurate preoperative characterization is essential for surgical planning and toric intraocular lens (IOL) selection. Data from Southern European populations remain limited.

**Methods:**

We conducted a retrospective cohort study including 600 eyes from 306 consecutive cataract patients aged ≥ 40 years undergoing preoperative biometry at a tertiary university hospital in Southern Italy. Axial length (AL), anterior chamber depth (ACD), keratometric power (K), and corneal astigmatism (CA) were measured using partial coherence interferometry.

**Results:**

The mean patient age was 72.5 ± 8.5 years. The mean AL, ACD, and average K were 23.5 ± 1.6 mm, 3.1 ± 0.5 mm, and 43.9 ± 1.5 D, respectively. The mean CA was 1.02 ± 0.76 D. Notably, 39.8% of eyes had CA ≥ 1.0 D and 9.3% had CA ≥ 2.0 D. Eyes with AL > 26 mm accounted for 7.1% of the cohort.

**Conclusion:**

A substantial proportion of cataract patients in Southern Italy present with clinically significant corneal astigmatism, highlighting the importance of systematic preoperative screening. These findings support the routine consideration of toric IOLs to optimize refractive outcomes and meet the rising expectations of cataract surgery patients.

## Introduction

Cataract remains the leading cause of visual impairment worldwide, accounting for a substantial proportion of reversible blindness despite continuous advances in surgical techniques and intraocular lens (IOL) technology. Modern cataract surgery is increasingly considered a refractive procedure, with patients expecting spectacle independence and high-quality uncorrected visual acuity postoperatively (1).

Corneal astigmatism plays a critical role in determining visual outcomes. Even low levels of residual astigmatism may compromise uncorrected distance visual acuity and patient satisfaction, particularly in the era of premium and toric IOLs. Previous epidemiological studies have reported that 30%–40% of cataract candidates present with ≥1.0 diopter (D) of corneal astigmatism, underscoring the need for systematic preoperative evaluation and tailored refractive strategies (2, 3).

Although several large-scale studies have investigated biometric characteristics and the prevalence of corneal astigmatism across different populations in Europe and Asia, (2–7), data from Mediterranean cohorts–and particularly Southern Italy–are limited. Given the potential influence of demographic and ethnic factors on biometric parameters, region-specific data are essential for refining surgical planning and guiding toric IOL adoption.

The aim of this study was to analyze ocular biometric characteristics and the prevalence of corneal astigmatism in a cohort of cataract surgery candidates from Southern Italy. By identifying the distribution of biometric parameters and the proportion of patients with clinically relevant astigmatism, we sought to provide evidence to support optimized preoperative screening and refractive management strategies in this population.

## Materials and methods

This study comprised consecutive cataract candidates scheduled for phacoemulsification and foldable intraocular lens (IOL) implantation at a University hospital in Bari (Italy). The study adhered to the principles outlined in the Declaration of Helsinki. Given that this was a retrospective study utilizing cumulative data with no potential for identifying individual patient information, ethical board approval was not required. The inclusion criteria were cataract and age 40 years or older. Exclusion criteria included corneal or ocular surface pathologies, previous corneal or intraocular surgery, history of ocular trauma or inflammation. All the patients were stratified into 5 groups on the basis of age as follows: 40–50 years, 51–60 years, 61–70 years, 71–80 years, and 81 years and older.

The primary outcome of the study was the analysis of the distribution of pre-operative corneal astigmatism. Secondary outcome was the evaluation of the distribution of ocular biometric parameters in the study population.

Data regarding axial length (AL), anterior chamber depth (ACD), keratometric power (K), and corneal astigmatism (CA) were determined using partial coherence laser interferometry (PCI) with an infrared diode laser at a 780 nm wavelength (IOLMaster 500, Carl Zeiss Meditec AG). AL was measured as the distance from the tear film to the retinal pigment epithelium; ACD was assessed as the distance from the anterior corneal surface to the anterior lens surface. Corneal power was measured in 2 meridians; that is, flat keratometry (K1) and steep K (K2). The *K*-value was the mean of K1 and K2. All biometric parameters (AL, ACD, K1, K2 and corneal astigmatism) were automatically measured using the integrated software of the IOLMaster 500 (Carl Zeiss Meditec AG), without any manual adjustment. The refractive index value used by PCI was 1.3375.

All patients included in this study underwent a standardized protocol for ocular surface optimization prior to biometric measurements. This protocol consisted of a 2- to 4-weeks course of artificial tears, combined with eyelid hygiene using wipes and warm compresses. These measures aimed to stabilize the tear film and reduce ocular surface inflammation, thereby minimizing measurement variability caused by dry eye or other surface disorders. Accurate assessment of corneal astigmatism is critical, as ocular surface irregularities can introduce “noise” that affects keratometry and topography readings, potentially leading to refractive surprises postoperatively (8). By optimizing the ocular surface, we sought to enhance the reliability of preoperative astigmatism measurements and ensure more precise surgical planning. Contact lens wearers discontinued lenses for at least 1 week (soft lenses) or 3 weeks (rigid gas-permeable lenses) prior to biometry to avoid keratometric variability.

Prior to inferential analyses, the distribution of continuous variables was assessed for normality using the Shapiro–Wilk test, supported by visual inspection of Q–Q plots. Homogeneity of variance across age groups was evaluated using Levene’s test. Since assumptions for parametric testing were met, comparisons among groups were performed using one-way analysis of variance (ANOVA). Pearson correlation coefficients were calculated to assess linear associations between biometric parameters. Statistical significance was set at *p* < 0.05.

## Results

A total of 600 phakic eyes from 306 consecutive cataract patients were included in the analysis. The mean patient age was 72.45 ± 8.48 years (range: 43–89), with the largest proportion (34.2%) falling into the 71–80 year group. Table 1 summarizes the distribution of biometric parameters across the entire sample.

**TABLE 1:** Medium age and ocular biometric parameters.

	Medium ± SD	Min-max
Age (years)	72.45 ± 8.48	43–89
40–50 years (*n* = 4)		
51–60 years (*n* = 30)		
61–70 years (*n* = 112)		
71–80 years (*n* = 205)		
81 or more years (*n* = 71)		
AL (mm)	23.53 ± 1.58	20.64–29.72
ACD (mm)	3.12 ± 0.48	1.61–5.15
K1 (D)	43.39 ± 1.49	39.89–47.4
K2 (D)	44.45 ± 1.54	41.26–48.7
avg K (D)	43.95 ± 1.5	40.32–47.92
CA (D)	1.02 ± 0.76	0.00–4.32

AL, axial length; ACD, anterior chamber depth; K, keratometric power of flat axis (K1) steep axis (K2) and average power (avg K); CA, corneal astigmatism; D, diopter; mm, millimeter; SD, standard deviation.

The mean AL was 23.53 ± 1.58 mm (range: 20.64–29.72 mm), and ACD averaged 3.12 ± 0.48 mm (range: 1.61–5.15 mm). Keratometric power showed a mean of 43.39 ± 1.49 D on the flat axis and 44.45 ± 1.54 D on the steep axis, with a Kavg of 43.95 ± 1.50 D. The CA was 1.02 ± 0.76 D, with values ranging from 0.00 to 4.32 D. CA distribution was as follows:

- 0–0.24 D: 7.3%
- 0.25–0.74 D: 37.4%
- 0.75–0.99 D: 17.5%
- 1.00–1.49 D: 17.5%
- 1.50–1.99 D: 9.7%
- 2.00–2.49 D: 5.3%
- 2.50–2.99 D: 3.9%
- ≥3.00 D: 1.5%

Notably, approximately 40% of eyes had a preoperative CA of ≥1.0 D, and over 9% had CA ≥ 2.0 D, underscoring the clinical relevance of toric IOL implantation in this population. Corneal astigmatism showed a borderline increasing trend across age groups (one-way ANOVA: *F* = 2.39, *p* = 0.052). The proportion of eyes with CA ≥ 1.0 D did not significantly differ among age strata (χ^2^ = 4.41, *p* = 0.354). No sex-related differences in CA magnitude were observed (males: 0.99 ± 0.62 D; females: 0.99 ± 0.75 D; *p* = 0.994).

A subset of eyes (7.11%) had an AL exceeding 26 mm, suggesting a small but significant proportion with high axial myopia. The biometric variability observed across the population highlights the necessity for individualized IOL selection strategies.

**FIGURE 1:**
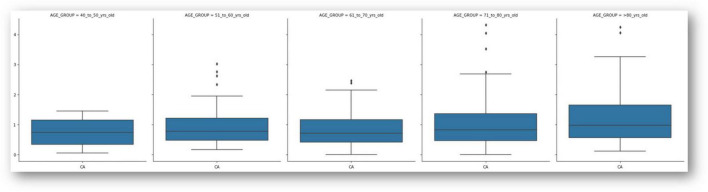
Box-plot of corneal astigmatism values in the 5 age groups. The bold lines inside the “boxes” represent the median, the upper and lower bounds of the rectangles represent the first and third quartiles, the “mustaches” represent the minimum and maximum values. Asterisks represent outliers. CA, corneal astigmatism.

In the age-stratified analysis, presented in Figure 1, a trend was observed with slightly increasing astigmatism in the older age groups, although the differences were not statistically analyzed in this preliminary study. Box plots of CA across the five age groups demonstrate increasing interquartile spread and maximum values with advancing age.

**FIGURE 2:**
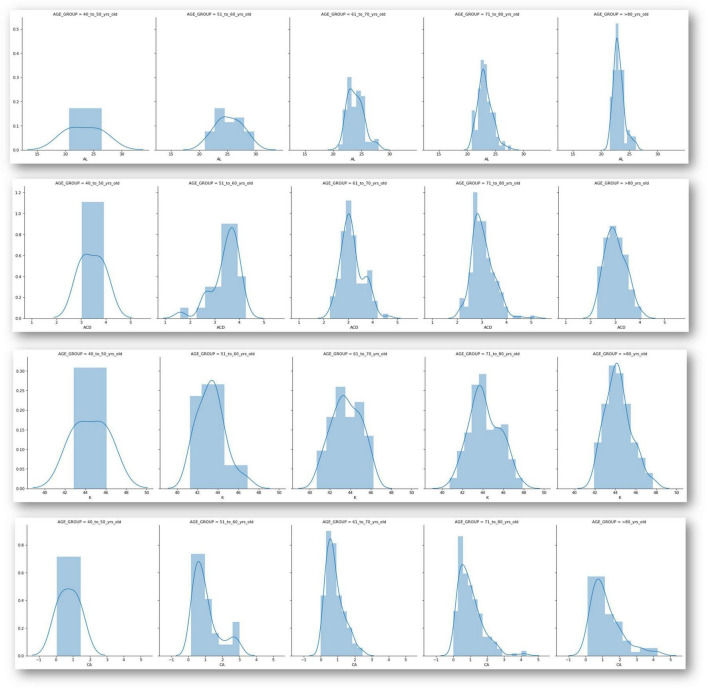
Distribution analysis of main parameters divided among different age groups. AL, axial length; ACD, anterior chamber depth; K, keratometry; CA, corneal astigmatism.

**FIGURE 3:**
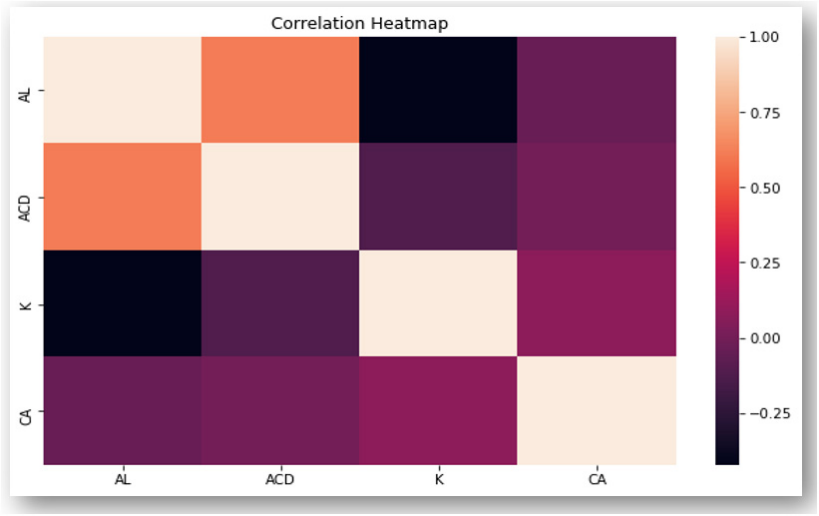
Correlation heatmap which displays the correlation between multiple variables as a color-coded matrix. AL, axial length; ACD, anterior chamber depth; K, keratometry; CA, corneal astigmatism.

Figure 2 displays the distribution of all biometric parameters across age groups. Pearson correlation analysis revealed: AL–ACD *r* = 0.624, *p* < 0.001; AL–Kavg *r* = −0.403, *p* < 0.001; K2–CA *r* = 0.268, *p* < 0.001; Age–CA *r* = 0.152, *p* = 0.029; AL–CA *r* = −0.032, *p* = 0.643. The correlation matrix in Figure 3 shows moderate positive correlations between axial length and anterior chamber depth, and weak associations between keratometric values and CA, warranting further multivariate investigation in future work.

A *post hoc* power analysis was performed to evaluate the adequacy of the sample size. With 600 eyes included, the study had >80% power to detect small correlation coefficients (*r* ≥ 0.12–0.15) at a two-sided α level of 0.05, indicating sufficient power to identify clinically relevant associations.

## Discussion

This study evaluated the distribution of ocular biometric parameters and the prevalence of corneal astigmatism in a Southern Italian cataract population. Consistent with prior reports, approximately 40% of eyes exhibited preoperative corneal astigmatism greater than 1.0 D, with 10% exceeding 2.0 D. The refined astigmatism stratification demonstrates that more than half of our cohort presented ≥0.75 D, and nearly three quarters ≥0.50 D. These thresholds are clinically relevant, given that several contemporary studies demonstrate measurable UDVA degradation and reduced spectacle independence even for residual astigmatism between 0.25 and 0.50 D. These findings reaffirm that a significant proportion of cataract patients harbor astigmatism levels that warrant targeted correction to optimize postoperative visual outcomes. Our findings support the consideration of toric IOLs in cataract candidates with clinically meaningful astigmatism, while recognizing that cost-effectiveness and postoperative refractive outcomes were not evaluated in this study.

The pursuit of optimal uncorrected visual acuity and spectacle independence following cataract surgery has intensified clinical focus on the correction of residual refractive errors, particularly astigmatism. While traditionally a residual astigmatism threshold of 0.75 D was considered clinically significant, emerging evidence demonstrates that even lower levels–between 0.25 and 0.50 D–can adversely affect uncorrected distance visual acuity (UDVA) and patient satisfaction. Schallhorn et al., in a robust cohort study of over 17,000 eyes, demonstrated that eyes with residual astigmatism in this lower range were significantly less likely to achieve 20/20 UDVA compared to eyes without residual astigmatism, across both monofocal and multifocal IOLs (9). This highlights the necessity for meticulous astigmatic correction to meet the rising expectations of modern cataract patients, particularly in the era of premium intraocular lenses.

Moreover, the axis of residual astigmatism critically influences postoperative visual quality. Against-the-rule (ATR) and oblique astigmatism are associated with greater visual disturbances and are more challenging to correct accurately with standard toric IOLs, which tend to be optimized for with-the-rule (WTR) astigmatism (10). Failure to adequately address ATR or oblique astigmatism can lead to suboptimal refractive outcomes despite lens implantation, emphasizing the importance of thorough preoperative characterization of astigmatism axis and personalized surgical planning.

Several surgical options exist to manage corneal astigmatism in cataract patients. Toric IOLs provide a precise and effective method of correcting moderate to high astigmatism and can be particularly beneficial for ATR and oblique axes when properly aligned (11). Although, previous studies shown that the adoption of toric IOLs remained low, i.e., 4.5% of whom with significant corneal astigmatism and the change was slow (0% in years 2010–2014 vs. 1.8% in years 2015–2018) in patients older than 81 years, (12) less prone to pay for toric IOL with advanced age (13). Corneal astigmatism in patients who are candidates for cataract surgery can also be treated intraoperatively by making relaxing limbal incisions. However, the results obtained from these incisions can sometimes be inaccurate and unpredictable due to variations in wound healing and surgical technique. An alternative or adjunctive approach involves parallel corneal penetrating incisions, strategically placed to reduce astigmatism by precisely altering corneal curvature. Despite this, the effectiveness of these incisions can still vary depending on the incision depth, length, and location, as well as individual corneal biomechanics, sometimes leading to under- or over-correction. Furthermore, the location, size, and architecture of the primary cataract incision can influence the final astigmatic outcome, with superior incisions generally inducing more ATR shift compared to temporal approaches (14). Postoperative refractive enhancements, such as LASIK, intracorneal ring segments or other laser vision correction procedures, remain viable options for residual astigmatism but add complexity and are not suitable for all patients (15, 16). When considering toric IOL implantation, potential contraindications should be carefully ruled out, including irregular astigmatism or corneal ectasia and significant zonular weakness.

In our population, the significant prevalence of astigmatism greater than 0.75 D underscores the potential demand for toric IOLs and complementary refractive strategies. Incorporating advanced diagnostic tools such as vector planning and corneal topography into preoperative evaluation enhances surgical planning and optimizes refractive predictability. Furthermore, ensuring ocular surface optimization prior to biometry–through artificial tears, warm compresses, and lid hygiene–reduces measurement variability caused by dry eye or ocular surface disease, thereby improving the accuracy of astigmatism assessment.

Limitations of this study include its retrospective design and the absence of detailed subgroup analyses, such as sex-based comparisons or evaluation of astigmatism axis distribution. Additionally, the lack of follow-up data precludes correlation with postoperative outcomes. Nonetheless, the strength of the study lies in its relatively large sample size and the use of standardized measurement protocols, ensuring internal consistency.

In conclusion, this study confirms that a substantial subset of cataract patients exhibits corneal astigmatism requiring correction for optimal visual outcomes. Given the impact of even low levels of residual astigmatism on postoperative vision and patient satisfaction, a tailored approach combining toric IOL implantation, corneal incisions, and postoperative refractive options is warranted. Such individualized strategies are essential to meet the evolving expectations for spectacle independence and high-quality vision in cataract surgery.

## Data Availability

The raw data supporting the conclusions of this article will be made available by the authors, without undue reservation.
